# Cryopreserved, Thin, Laser-Etched Osteochondral Allograft maintains the functional components of articular cartilage after 2 years of storage

**DOI:** 10.1186/s13018-020-02049-y

**Published:** 2020-11-11

**Authors:** Carolyn B. Rorick, Jordyn A. Mitchell, Ruth H. Bledsoe, Michael L. Floren, Ross M. Wilkins

**Affiliations:** Innovation Department, AlloSource, 6278 S Troy Circle, Centennial, CO 80111 USA

**Keywords:** Articular cartilage repair, Osteochondral allograft, Chondrocytes, Cryopreservation, Microfracture, MSC signaling, Chondrogenesis

## Abstract

**Background:**

Despite improvements in treatment options and techniques, articular cartilage repair continues to be a challenge for orthopedic surgeons. This study provides data to support that the 2-year Cryopreserved, Thin, Laser-Etched Osteochondral Allograft (T-LE Allograft) embodies the necessary viable cells, protein signaling, and extracellular matrix (ECM) scaffold found in fresh cartilage in order to facilitate a positive clinical outcome for cartilage defect replacement and repair.

**Methods:**

Viability testing was performed by digestion of the graft, and cells were counted using a trypan blue assay. Growth factor and ECM protein content was quantified using biochemical assays. A fixation model was introduced to assess tissue outgrowth capability and cellular metabolic activity in vitro. Histological and immunofluorescence staining were employed to confirm tissue architecture, cellular outgrowth, and presence of ECM. The effects of the T-LE Allograft to signal bone marrow-derived mesenchymal stem cell (BM-MSC) migration and chondrogenic differentiation were evaluated using in vitro co-culture assays. Immunogenicity testing was completed using flow cytometry analysis of cells obtained from digested T-LE Allografts and fresh articular cartilage.

**Results:**

Average viability of the T-LE Allograft post-thaw was found to be 94.97 ± 3.38%, compared to 98.83 ± 0.43% for fresh articular cartilage. Explant studies from the in vitro fixation model confirmed the long-term viability and proliferative capacity of these chondrocytes. Growth factor and ECM proteins were quantified for the T-LE Allograft revealing similar profiles to fresh articular cartilage. Cellular signaling of the T-LE Allograft and fresh articular cartilage both exhibited similar outcomes in co-culture for migration and differentiation of BM-MSCs. Flow cytometry testing confirmed the T-LE Allograft is immune-privileged as it is negative for immunogenic markers and positive for chondrogenic markers.

**Conclusions:**

Using our novel, proprietary cryopreservation method, the T-LE Allograft, retains excellent cellular viability, with native-like growth factor and ECM composition of healthy cartilage after 2 years of storage at − 80 °C. The successful cryopreservation of the T-LE Allograft alleviates the limited availably of conventionally used fresh osteochondral allograft (OCA), by providing a readily available and simple to use allograft solution. The results presented in this paper supports clinical data that the T-LE Allograft can be a successful option for repairing chondral defects.

**Supplementary Information:**

The online version contains supplementary material available at 10.1186/s13018-020-02049-y.

## Background

Chondral and osteochondral defects are a common problem in orthopedics and can cause patients to suffer from pain, functional impairment, and poor quality of life [1–3]. In particular, chondral defects present a difficult clinical challenge to physicians and can predispose patients to developing degenerative joint disease, which can be debilitating especially in active adults [3, 4]. Human hyaline cartilage is comprised of a unique profile of chondrocytes (cells), matrix proteins (signals), and tissue architecture (scaffold), which together this advanced structure-function relationship imparts critical properties to the joint spaces such as cushioning and lubrication [5]. Unlike many tissues in the body, cartilage lacks a vascular supply network and therefore has a very limited self-repair capacity. Due to this inability to spontaneously repair, initially small cartilage lesions can progress into extensive chondral damage over time, necessitating surgical correction [6]. While several surgical options have emerged with the goal to reduce patient pain and restore the function of injured articular cartilage, current treatments are limited by the lack of available allograft options as well as from poor graft integration outcomes [7].

The current standard in cartilage repair for full thickness chondral lesions, due to its extensive clinical history and acceptable long-term success rates, is fresh osteochondral allograft (OCA) transplantation, where articular cartilage and underlying bone is transplanted from a cadaveric donor into the defect site [8, 9]. Due to the limited number of donors that meet the criteria for fresh cartilage donation and required size matching, these grafts are in short supply limiting accessibility to clinicians and patients. Furthermore, availability of fresh OCA grafts is also limited by a short shelf life; for instance, OCA grafts stored at 4 °C have an average shelf life of 28 days. This is due to suboptimal storage methods which can reduce cartilage viability below the 70% minimum needed for successful clinical outcomes [10]. Moreover, standard serological and microbial screening requires a minimum of 14 days additional storage, further reducing the window for successful graft implantation [11].

In the absence of an allograft, microfracture is a common treatment for small chondral defects with minimal subchondral bone damage. Microfracture is a technique which introduces small fractures into underlying bone, the introduction of these microinjuries at the site of a cartilage defect is thought to signal the migration of regenerative cells from the exposed underlying bone marrow, inducing new tissue formation [12]. However, post-operative follow-up studies indicate that microfracture does not provide successful long-term clinical outcomes due to the regenerated cartilage structure composed primarily of fibrocartilage which is more inclined to degradation as opposed to hyaline cartilage [13, 14]. Further studies indicate that poor regeneration of cartilage following microfracture procedure might be due to a lack of proper chondrogenic signaling, leading to improper differentiation of the stem cells migrating out of the bone [15]. To address this issue, several alternative procedures have been tested, including filling the cartilage defect site with a living cell allograft which provides an intact tissue matrix with chondrocytes to properly direct tissue regeneration [13]. Living cell allografts may provide the necessary microenvironment (cells, signal, scaffold) to help stem cells released by microfracture properly differentiate and produce a more hyaline-like cartilage [13, 16, 17]. Therefore, the ability to have an off-the-shelf cartilage allograft which integrates cells, signals, and tissue scaffold with excellent storage capabilities would be a remarkable addition to the osteochondral therapeutic landscape.

To address the limitations of current treatment options, it is critical to examine alternative tissue processing strategies that supply the essential viable cells, growth factor signaling, and extracellular matrix scaffold required for successful cartilage defect repair. Here, we present an additional option for cartilage restoration and repair, the Cryopreserved, Thin, Laser-Etched Osteochondral Allograft (T-LE Allograft) (AlloSource®, Centennial, CO, USA), which contains these three essential components necessary for successful clinical outcomes. The T-LE Allograft is a minimally manipulated, living cell, thin osteochondral allograft with a cryopreserved 2-year shelf life. Laser-etching promotes cellular outgrowth and flexibility to allow for repair of chondral defects in various joints [18]. A novel procedure for cryopreservation allows the T-LE Allograft to retain the functional components of traditional fresh OCAs, while extending the shelf life and availability to patients and clinicians. In this paper, we characterize and compare the T-LE Allograft to freshly recovered cartilage tissue to demonstrate the benign nature of our processing method, which protects the native profiles of cell viability, matrix proteins, and tissue scaffolding, all of which are essential components for successful clinical outcomes for articular cartilage defect repair.

## Methods

### Tissue collection and T-LE Allograft preparation

Articular cartilage was obtained from human donors deemed eligible for tissue donation for research purposes (Supplementary Table 1). Fresh cartilage tissue samples and T-LE Allograft tissue was obtained from 36- to 39-year-old male and female research consented cadaveric human donors which were recovered within 72 h of death; articular cartilage was harvested from uncompromised regions of weight-bearing joint surfaces from the femur, tibia, and talus. Once collected, all tissue samples were placed in a sterile container with Chondrocyte Growth Medium (CGM) (Cell Applications, San Diego, CA, USA) and used immediately or stored at 4 °C until further processing.

To prepare the T-LE Allograft samples, articular cartilage was shaved to a 1-mm thickness and punched into disks between 9 and 20 mm in diameter, followed by a proprietary laser-etching and cryopreservation process prior to being stored at − 80 °C for 2 years until further testing. For fresh articular cartilage, tissue was processed no more than 72 h after death for analysis.

### Viability testing

For viability testing, twelve T-LE Allografts cryopreserved for 2 years at − 80 °C and nine fresh articular cartilage specimens were digested in a collagenase digestion solution consisting of CGM, 1 mg/ml collagenase type I (MediaTech, Manassas, VA, USA) and 2 mg/ml collagenase type II (GibCO, Gaithersburg, MD, USA) overnight at standard conditions (37 °C; 5% CO_2_). The collected tissue digests were run through a 40-μm cell strainer to eliminate particulate debris and spun down at 500×*g* for 10 min to produce a cell pellet. The cell concentrate produced was then stained with trypan blue, an exclusion dye, at a 1:1 ratio (Invitrogen, Carlsbad, CA, USA) and counted using an automated cell counter (Invitrogen, Carlsbad, CA, USA). Viability results were averaged for each sample and test group, with the standard deviation (SD) reported for each group.

For live/dead staining a 3-mm biopsy punch was taken from a T-LE Allograft and placed into 0.5 mL of phosphate-buffered saline (PBS) (Mediatech, Manassas, VA, USA), with 0.5 μL of both calcein AM and ethidium homodimer-1 (Invitrogen, Carlsbad, CA, USA) and added to a 24-well plate, which was then wrapped in foil to protect from light and incubated for 30 min at room temperature. Samples were then imaged using a Nikon D-Eclipse C1 Laser Scanning Confocal Microscope (Nikon, Tokyo, Japan).

### In vitro fixation outgrowth assay and metabolic testing

To evaluate the capacity for T-LE Allografts to retain metabolic activity in the presence of a fixation method, an in vitro model was developed to monitor graft tissue outgrowth and metabolism over several time points. First, thin layers of TISSEEL (Baxter, Deerfield, IL, USA) fibrin glue, a standard method of graft fixation in a clinical setting (Supplementary Figure 1A), were placed on the bottoms of a 24-well culture plate followed by promptly placing the tissue graft on the TISSEEL surface and culturing for several week intervals [19]. T-LE Allografts cryopreserved for 2 years at − 80 °C and fresh non-cryopreserved T-LE Allografts 14 days post date of death (stored at 4 °C in media) were utilized to report cellular outgrowth from the graft into the TISSEEL layer in vitro. The metabolic activity of chondrocyte outgrowth and proliferation of the graft was assessed using PrestoBlue™ Dye (Life Technologies, Carlsbad, CA, USA) at 0, 3, 6, 9, and 12 weeks post-fixation. PrestoBlue dye was added at a 1:10 ratio and the samples were incubated for 3 h under standard conditions. Chondrocytes used for the standard curve of the PrestoBlue assay were produced through a digestion of fresh cartilage tissue in a collagenase digestion solution overnight. These cells were cultured for at least 1 week and were used within 6 passages. A 100-μL aliquot of each sample and known standard was placed in triplicate into a 96-well plate and read using a plate reader at 535 and 615 nm. Fluorescent readings were recorded using a Synergy H1 Hybrid Reader (Biotek, Winooski, VT, USA) and quantified using the linear regression fit equation produced from the standard curve.

The viable cell count for each donor was averaged from multiple samples. The donors were then grouped based on preservation method, then the average of the fresh T-LE Allografts (3 donors) and the average of the cryopreserved T-LE Allografts (3 donors) were visualized in a bar graph. The standard deviation was calculated from all data points for each group and was used to calculate the standard error of the mean (SEM) which is shown as plus or minus error bars for each group at each time point.

### Fixation assay histology and immunofluorescent (IF) imaging

At 12 weeks culture in the fixation assay, explanted T-LE Allografts were fixed with 10% neutral buffered formalin (Richard Allen Scientific, Kalamazoo, MI, USA) for 48 h, processed, embedded, and cut. The tissue cross-sections were placed on glass histology slides and stained for Safranin-O (IHC World, Woodstock, MD, USA) and hematoxylin and eosin (H&E) (VWR, Radnor, PA, USA) according to the manufacturer’s protocol. The explanted samples were also stained for immunofluorescent imaging with primary-conjugated antibody Collagen-II FITC (Life Sciences Bio, Seattle, WA, USA) and primary-unconjugated antibody Aggrecan (R&D Systems, Minneapolis, MN, USA) samples were counterstained with a PE-conjugated secondary antibody Mouse Anti-Human IgM Fc Fragment (Novus Biologicals, Centennial, CO, USA). The fixed histology sections were blocked with 10% fetal bovine serum (FBS) (Omega Scientific, Tarzana, CA, USA) in PBS for 1 h. The primary antibodies were added to the samples at a concentration of 1:200 (Collagen II) and 1:100 (Aggrecan) in 1.5% FBS/PBS to incubate overnight at 4 °C and protected from light. The samples were washed and secondary antibody was added at a concentration of 1:100 in 1.5% FBS/PBS and incubated for 2 h protected from light. The samples were then washed and imaged on a confocal microscope. Histology samples were imaged with a Nikon Eclipse Ci Microscope (Nikon, Tokyo, Japan). Immunofluorescent imaging was performed using a Nikon D-Eclipse C1 Laser Scanning Confocal Microscope (Nikon, Tokyo, Japan).

### Growth factor and sulfated glycosaminoglycan (sGAG) testing

T-LE Allografts cryopreserved for 2 years and fresh articular cartilage were incubated in CGM with 2% Antibiotic-Antimycotic (GibCO, Gaithersburg MD, USA) for 1 week under standard conditions. Following the cell culture, the cell media supernatant was collected and stored at − 80 °C for testing. Tissue was homogenized with Cell Lysis Buffer (RayBiotech, Norcross, GA, USA) and Protease Inhibitor Cocktail (Thermo Scientific, Waltham, MA, USA). Homogenized samples were sonicated and placed on ice for 2 h. Homogenates were passed through a 100-μm cell strainer and centrifuged at 12,000×*g* for 10 min. Tissue lysate was collected and stored at − 80 °C until further use.

For assessing growth factors, tissue lysates and cell culture supernatants from T-LE Allograft and fresh articular cartilage tissue were analyzed for the activity of immunosorbent antibodies to detect and quantify the protein content of Bone Morphogenic Protein-7 (BMP-7), Transforming Growth Factor-β1 (TGF-β1), Proteoglycan-4 (PRG-4), and Basic Fibroblast Growth Factor (b-FGF). T-LE Allografts growth factor profiles were evaluated using ELISA kits (Blue Gene Biotech, Shanghai, China) for TGF-β1, (Wuhan Fine Biotech Co., Ltd. Wuhan, China) for BMP-7, (Cusabio, Houston, TX, USA) for PRG-4 and (R&D Systems, Minneapolis, MN, USA) for b-FGF. ELISAs were performed per the manufacturer’s protocol and the results were read at 450 nm with a BioTek Synergy H1 Hybrid Reader. The data was normalized with the weight and volumes of the respective tissue homogenates and cell culture supernatants. For quantifying sGAG, cell culture supernatants were analyzed using a colorimetric assay which employs 1, 9-dimethylmethylene blue to detect sulfated glycosaminoglycans by providing a label for the sulfated polysaccharides of the proteoglycan. The culture supernatants were analyzed using a BioVision Blyscan Detection kit (BioColor Life Sciences, Carrickfergus, UK). The manufacturer’s protocol for the Blyscan Detection Kit was followed and the absorbance was read at 656 nm with a BioTek Synergy H1 Hybrid Reader. Standard curves were created for each assay and used to quantify the growth factors or matrix proteins in each sample, and data was then analyzed per manufacturer’s instructions.

After measuring the absorbance for each assay, replicate samples were averaged, and protein concentrations were calculated from the absorbance reading using each assay’s standard curve. The following types of curves were populated for each of the assays: Linear Regression Formula (BMP-7, b-FGF, sGAG), 4-PL Curve (PRG4, the 4-PL curve was constructed using the BioTeK Synergy H1 software and the concentrations were computed from the software), and a Logarithmic Curve (TGF-β). Each sample’s growth factor concentration was normalized using the sample’s initial weight and buffer dilution. A Dixon *Q* test was used to identify and eliminate outliers (*p* ≤ 0.05) in each sample group. The concentration values were then plotted on boxplots, and the sample number (*N*) of each group is shown below each boxplot. Due to availability of samples, not all donors could be analyzed for all assays. The *p* values were calculated using a Student’s *t* test, assuming unequal variance, comparing the mean of the fresh cartilage and the cryopreserved T-LE Allograft. For all analysis, a *p* value ≤ 0.05 was considered significant.

### BM-MSC migration and differentiation co-culture

Cryopreserved T-LE Allograft samples and fresh cartilage samples were co-cultured alongside bone marrow-derived mesenchymal stem cells (BM-MSC) to assess the allograft’s ability to signal BM-MSCs through the release of cytokines and growth factors (Supplemental Figure 1B, C). To extract BM-MSCs, vertebral bodies from a research consented donor were ground, washed, filtered, and concentrated to collect a cell pellet, which was then cryopreserved. Prior to use, the cryopreserved cell suspension was thawed in a 37 °C water bath. The cells were then lysed with ammonium chloride lysis buffer (BD Biosciences, San Jose, CA, USA) to remove red blood cells and filtered again through a 40-μm filter. The lysed and strained BM-MSCs were then cultured for the migration and differentiation assays.

For the migration assay, an 8.0-μm Transwell® insert (Thermo Fischer Scientific, Waltham, MA, USA) was seeded with 100,000 BM-MSCs and a 10-mm biopsy punch of the Cryopreserved T-LE Allograft or fresh cartilage was placed in the bottom of the well to avoid direct contact with the seeded cells. The cells and tissue were co-cultured for 24 h under standard conditions in Dulbecco’s modified Eagle medium (DMEM) (Corning, Corning, NY, USA) supplemented with 2% Antibiotic-Antimycotic (GibCO, Gaithersburg, MD, USA). After 24 h, the Transwell inserts were fixed with 10% neutral buffered formalin (Richard Allen Scientific, Kalamazoo, MI, USA), permeated with .01% Triton-X (Amresco Chemicals, Solon, OH, USA), unmigrated cells on the top side of the filter were wiped off with a cotton swab and the migrated cells were stained with May-Giemsa Stain (abcam, Cambridge, UK). The underside of the Transwell was imaged with a Leica DM IL LED (Leica Microsystems, Wetzlar, Germany) microscope for visual assessment of cellular migration.

For the differentiation assay, cryopreserved T-LE Allograft samples and fresh cartilage samples were also co-cultured alongside BM-MSC to assess the allograft’s ability to direct the MSCs into chondrogenic lineage. A 24-well plate was seeded with 100,000 BM-MSCs and incubated at standard conditions for 24 h to allow for attachment. Following BM-MSC attachment, a 6-mm biopsy punch of the cryopreserved T-LE Allograft or fresh cartilage was placed into the 8.0-μm Transwell insert (Thermo Fischer Scientific, Waltham, MA, USA) and placed into each well. The cells and tissue were co-cultured for 1 week under standard conditions in DMEM (Corning, Corning, NY, USA) supplemented with 2% Antibiotic-Antimycotic (GibCO, Gaithersburg, MD, USA) and 10% FBS (Omega Scientific, Tarzana, CA, USA). After 1 week, the plate was fixed with 10% neutral buffered formalin (Richard Allen Scientific, Kalamazoo, MI, USA) for 30 min, permeated with .01% Triton-X (Amresco Chemicals, Solon, OH, USA), stained with Alcian Blue (IHC World, Woodstock, MD, USA), and imaged with a Nikon Eclipse Ci Microscope (Nikon, Tokyo, Japan).

### Immunogenicity testing with flow cytometry

Cryopreserved T-LE Allograft samples and samples from fresh cartilage donors were collected for immunogenicity testing. The tissue samples were digested overnight under standard conditions in a collagenase digestion solution. Digestions were spun in a centrifuge at 500×*g* for 10 min, aspirated, and then resuspended in CGM. The cells were cultured under standard conditions in CGM until 80% confluency, after which the cells were trypsinized, counted, and diluted in a fluorescently activated cell sorting (FACS) buffer: 3% FBS (Omega Scientific, Tarzana, CA, USA), PBS (Mediatech, Manassas, VA, USA), 0.1% sodium azide (Sigma Aldrich, St. Louis, MO, USA). A concentration of 500,000 cells per 100 μl was labeled with 10 μl of the appropriate antibody or isotype control. The following labels were assessed: CD44-PE, CD44-PE Isotype Control, CD49a-Pacific Blue, CD49a-Pacific Blue Isotype Control, and CD45-FITC (BD Biosciences, San Jose, CA, USA). Samples were incubated for 30 min at 4 °C with the appropriate label and then spun in a centrifuge at 1500 rpm for 10 min. The supernatants were decanted, and the samples were resuspended in 500 μL of FACS buffer. Samples were run on the NovoCyte Flow Cytometer (ACEA Biosciences, Inc., San Diego CA, USA) with an overlay of the respective isotype control or negative control. A minimum of 100,000 events were collected for each specimen. The percent positive for each cluster of differentiation (CD) marker was calculated.

Quality control testing and compensation was performed on the NovoCyte Flow Cytometer prior to sample analysis. Debris was eliminated from the sample through FSC-H vs. SSC-H analysis of the samples. The FSC-H vs SSC-H plot also determined the size and granularity of the cells to ensure that the correct cell population was analyzed. The chondrocyte population for either the stained control or sample was gated using sequential gating methods for the specific expression marker, and a histogram was populated for the sample. The negative control samples were gated at 0.00% and the positive samples were measured as anything beyond that threshold on the *X*-axis. The sample and the isotype control were overlaid onto the same histogram. The percentage on the histogram represents the percent positive for that specific cluster of differentiation marker in the samples. A sample with a percentage of less than 1% was considered a negatable reading.

## Results

### Allograft preparation, structure, and viability testing

**Fig. 1:**
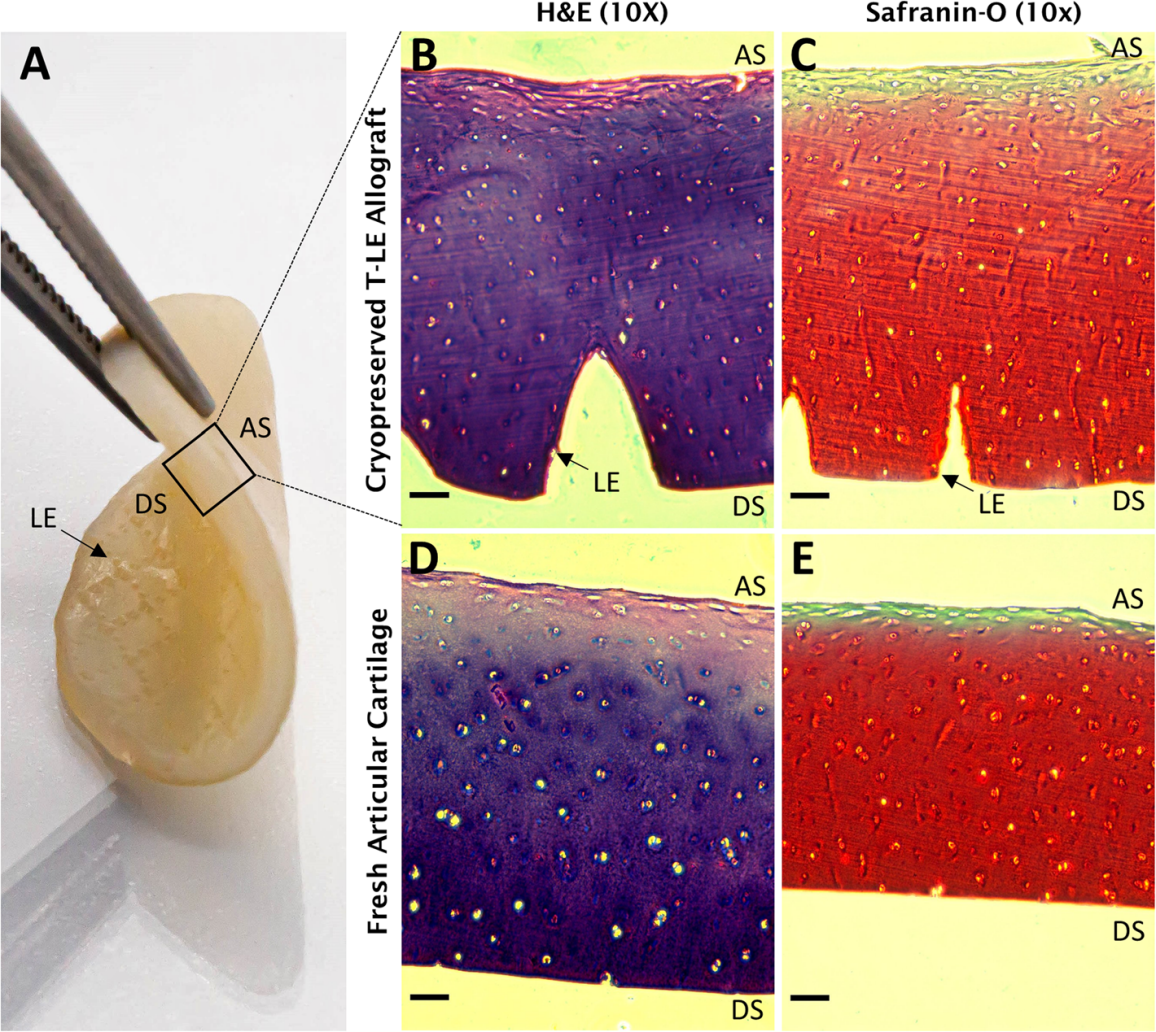
**a** The T-LE Allograft: the smooth articular surface (AS) and deep surface (DS) with laser-etching (LE) are pictured. **b**–**e** A structural comparison of the cross section of the cryopreserved T-LE Allograft (**b**, **c**) to fresh articular cartilage (**d**, **e**) with H&E (**b**, **d**) and Safranin-O (**c**, **e**) demonstrates the intact structure of native cartilage. Flattened, concentrated chondrocytes can be seen at the articular surface (AS) of the graft, with the orientation of chondrocytes elongating with the tissue depth. Laser-etching (LE) can be seen on the deep surface (DS) of the graft. Scale bar = 100 μm

The 2-year cryopreserved T-LE Allograft specimens were prepared from fresh articular cartilage using a proprietary method. This proprietary cryopreservation technique allows for a common cryoprotectant to penetrate the depth of the cartilage matrix using a rapid mass transfer technique; however, previously little was known about the preservation of structural and ECM components after this process. Histological staining shows (Fig. 1) that the processing of the T-LE Allograft does not disrupt the native structures as seen in unprocessed articular cartilage.

**Fig. 2:**
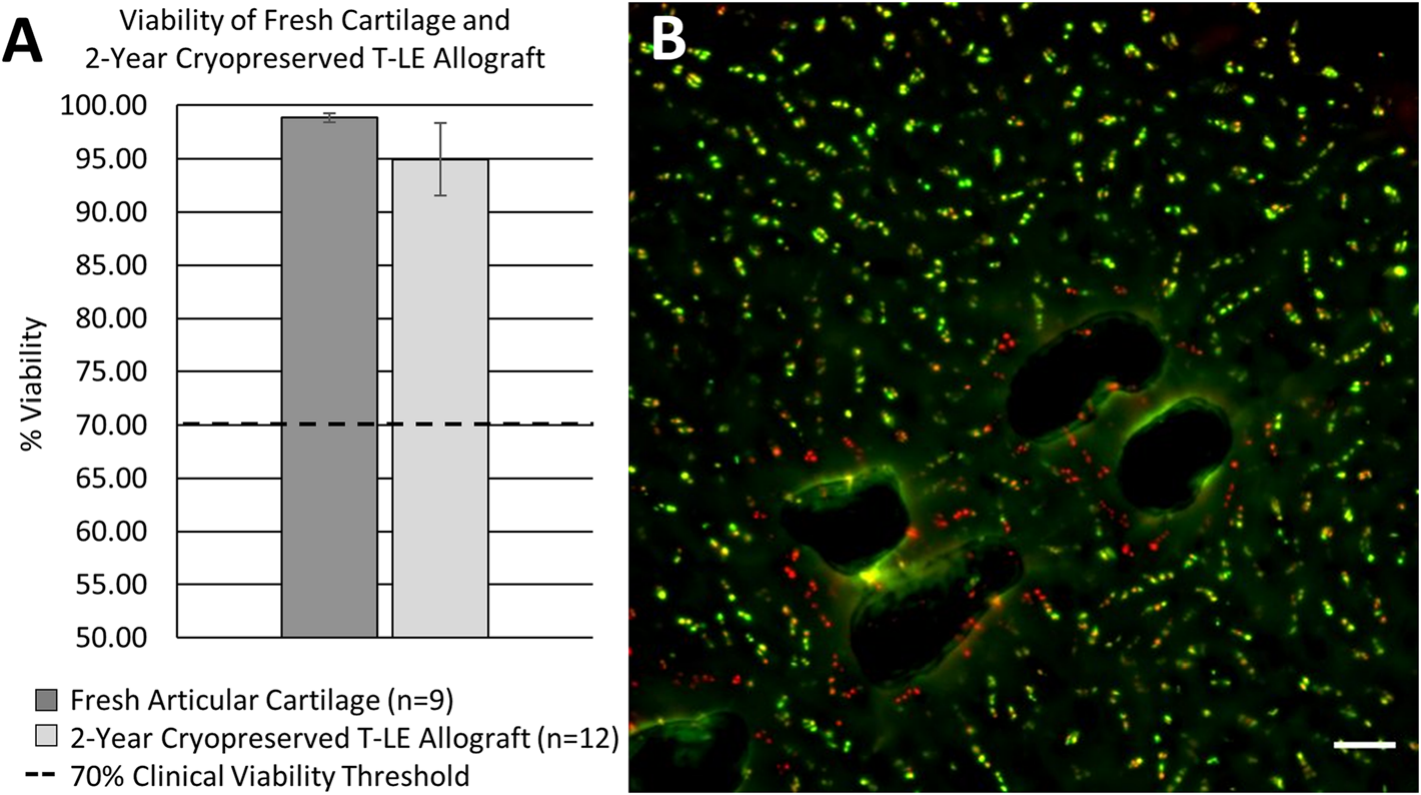
**a** The mean viability of the 2-year cryopreserved T-LE Allograft (*N* = 12) compared with fresh articular cartilage (*N* = 9). Error bars show each group’s standard deviation. The dotted line representing the 70% viability threshold required for successful clinical outcomes with OCA transplantation. **b** A live/dead (green, live cells; red, dead cells) stain of the T-LE Allograft (× 10) with visible laser perforations after cryopreservation. Scale bar = 100 μm

To access cell viability post-processing and cryopreservation, we first used a standard tissue digestion to isolate cells from the tissue grafts. After collection of the cell pellet, a trypan blue assay was used to quantify the cellular viability, as it has been shown to be a more reliable method of quantifying viability than live/dead immunofluorescent imaging [20]. Trypan blue is an exclusion dye method which stains dead cells, while live cells remain unstained, allowing for viability to be measured. We determined an average viability of 94.97 ± 3.38% for the T-LE Allograft grafts evaluated (*N* = 12), with a range of values from 86.67 to 98.67%. Comparably, fresh articular cartilage samples (*N* = 9) showed a viability of 98.83 ± 0.43%, with a range of 98.00 to 99.50% exemplified in Fig. 2a. Graft cell viability was further confirmed qualitatively by live/dead tissue staining (Fig. 2b). Together, these results suggest our proprietary graft laser-etching and cryopreservation process retains cell viability up to the preservation window of 2 years.

### Allograft outgrowth and metabolism using an in vitro fixation model

**Fig. 3:**
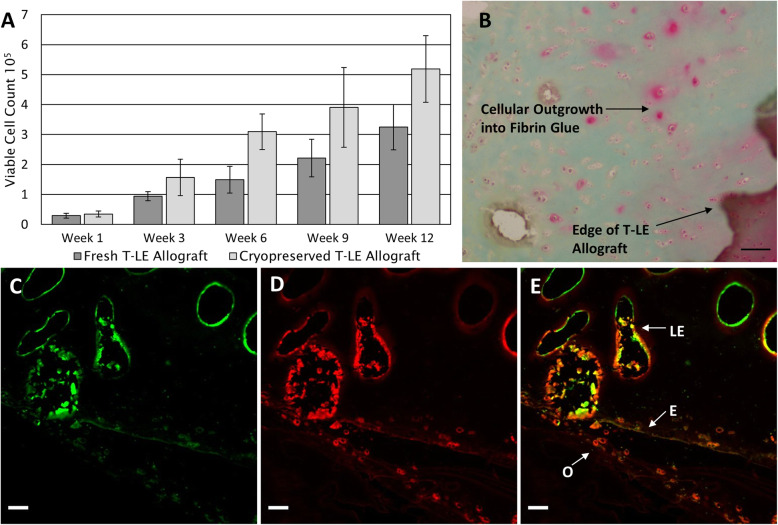
**a** Results from PrestoBlue metabolic assay. Graph represents the number of metabolically active cells throughout the duration of the explantation. A steady growth in viable cell count over time was observed for both fresh (14 days in media at 4 °C) and 2-year cryopreserved T-LE Allografts. Data presented as mean and standard error of the mean. **b** (× 20) Safranin-O stained outgrowth of cells from 12-week explanted cryopreserved T-LE Allograft into surrounding Fibrin glue. **c**–**e** Immunofluorescent staining results for explanted 2-year cryopreserved T-LE Allograft. Green fluorescence showing collagen II and red fluorescence showing aggrecan. The laser perorations (LE) can be seen in the T-LE Allograft, with cellular outgrowth (O) proliferating away from the graft edge (E), and into the surrounding fibrin glue. The T-LE Allograft maintains its extracellular matrix proteins, while metabolically active cells are secreting aggrecan into the surrounding fibrin glue. Scale bars = 100 μm

To demonstrate the capability of the T-LE Allograft to retain cellular outgrowth and metabolic activity upon implantation, an in vitro fixation model was developed using a common commercially available fixation fibrin glue, TISSEEL. Throughout the explantation of each T-LE Allograft onto a well plate containing fibrin glue, each sample was subjected to metabolic testing utilizing PrestoBlue viability dye. PrestoBlue is a non-destructive assay which quantifies the metabolic activity of each sample, thus allowing for the quantification of cells in the same sample over time. A linear growth of metabolically active cells was found to occur in both fresh and cryopreserved samples tested over the 12-week duration, as seen in Fig. 3a. This was evident as the confluency of the cells surrounding the graft visibly increases over time. We further confirmed the outgrowth and migration of cells from our T-LE Allografts visually using histology (Fig. 3b) as well as expression of ECM components using antigenic staining (Fig. 3c–e). The presence of ECM components native to cartilage, such as aggrecan and collagen II, were further confirmed through immunofluorescent (IF) imaging. The ability for viable cells within the T-LE Allograft to produce ECM components necessary for joint health and function is critical for the integration and longevity of implanted cartilage allografts.

### Growth factors and sGAG

**Fig. 4:**
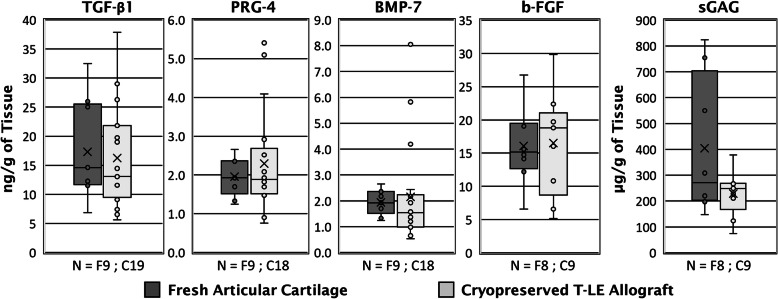
Growth factor and matrix protein concentrations in fresh cartilage and T-LE 2-year cryopreserved allograft samples. Boxplots show the median value (middle line), mean (x), and interquartile range (box) and whiskers extending to minimum and maximum values. Values exceeding 1.5 times the first or third quartile shown as single points. The sample number (N) of each group is shown below each boxplot. Cryopreserved T-LE Allografts exhibit no significant difference (*p* ≤ 0.05) in growth factor concentration and profile compared to fresh cartilage specimens

We next quantified the growth factor and matrix protein expression in fresh cartilage and cryopreserved T-LE Allograft samples. Cryopreserved T-LE Allografts showed no significant (*p* ≤ 0.05) reduction of growth factor proteins and sGAG content compared to fresh cartilage (Fig. 4). The retention of these factors within the T-LE Allograft indicates our novel cryopreservation method does not adversely affect the cell signaling capabilities or matrix protein secretion of the graft’s chondrocytes. This data suggests T-LE Allografts retain many of the important growth factors within the extracellular matrix even after 2 years cryopreservation.

### BM-MSC migration and differentiation

**Fig. 5:**
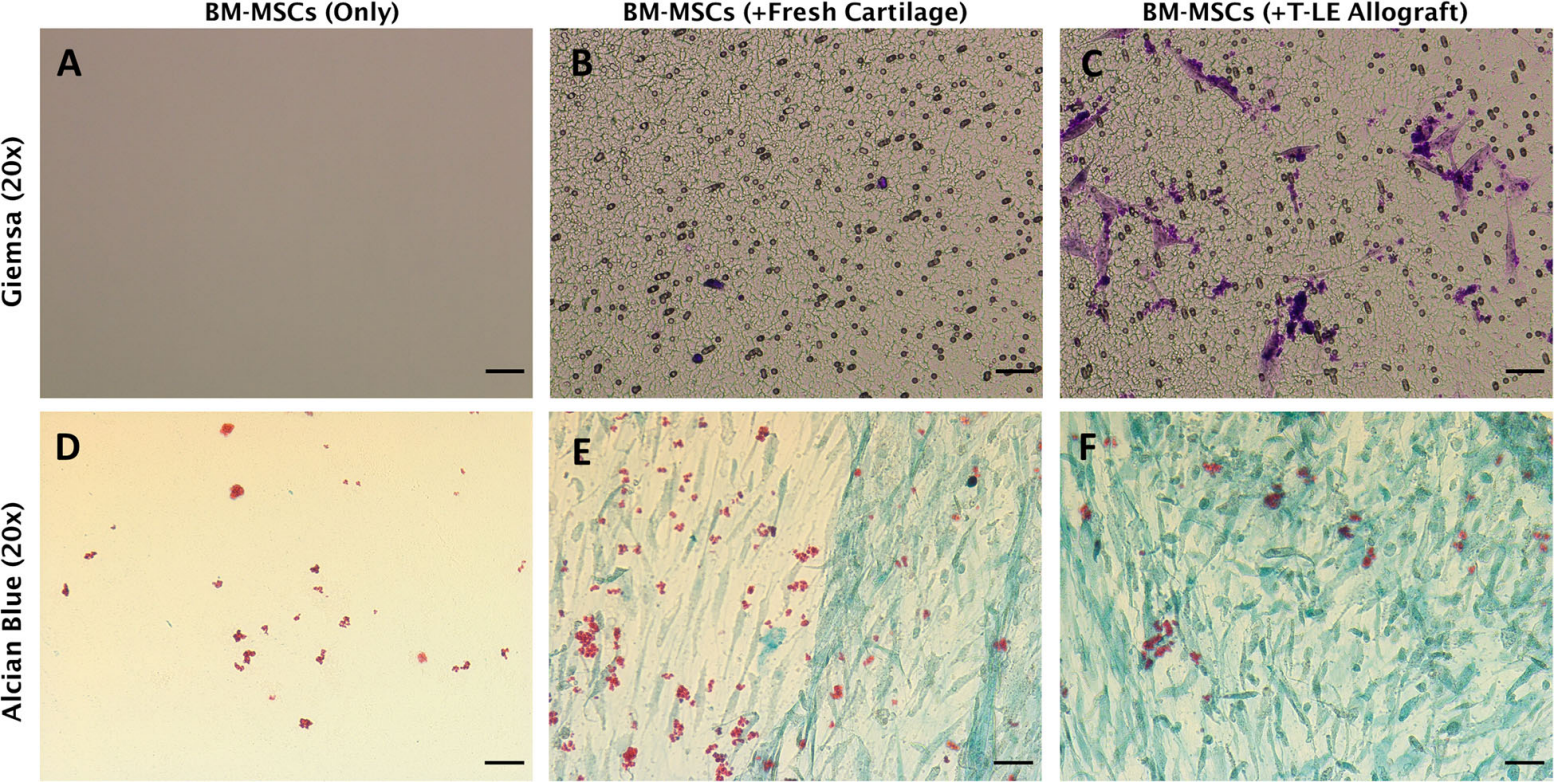
BM-MSC co-culture results for migration and differentiation. May-Giemsa stain (**a–c**) showing the presence of BM-MSCs after 24 h of migration in a Transwell plate. Both fresh (**b**) and T-LE Allograft (**c**) co-cultures showed increased migration compared to BM-MSCs cultured alone (**a**). Alcian Blue stain (**d**, **e**) reveals the glycosaminoglycan (GAG) content present in the deposited ECM from the BM-MSCs co-culture. Both fresh and T-LE Allograft co-cultures induced chondrogenic differentiation resulting in an increase in GAG content in the cells compared to BM-MSCs cultured alone. Scale bars = 100 μm

To ascertain the capacity for the T-LE Allograft to influence BM-MSCs through signaling pathways, we explored two co-culture experiments which provided information on BM-MSC migration and differentiation (Fig. 5). Histological staining of the BM-MSCs after co-culture with cryopreserved T-LE Allograft revealed the cryopreservation process retained the signaling capacity of the chondrocytes as observed through cell migration (Fig. 5a–c) and unique extracellular matrix deposition as observed by cells stained with Alcian Blue, as it stains for glycosaminoglycans excreted by chondrocytes (Fig. 5d–f). These combined results indicate that the T-LE Allograft is able to recruit BM-MSCs as well as induce chondrogenesis similar to fresh cartilage.

### Immunogenicity

**Fig. 6:**
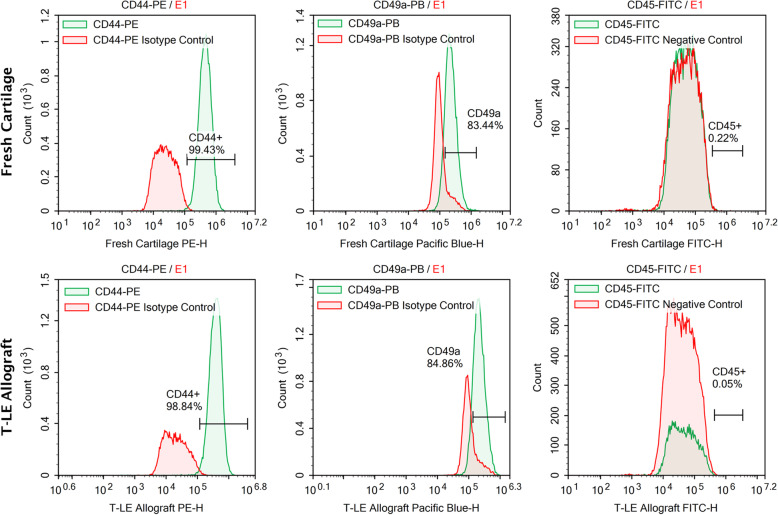
Chondrocytes from T-LE Allografts and fresh articular cartilage were recovered and expanded in culture. The chondrocytes were stained for chondrogenic markers CD49a, CD44, and immunogenic hematopoietic stem cell marker CD45. The chondrocytes show very little to no expression of CD45. The expanded chondrocytes show high expression of CD49a and CD44. The green and red histograms represent the isotype control and receptor expression profiles for each antibody investigated. The percent positive for each marker is represented on the respective histogram.

To determine the level of immunogenicity of our T-LE Allograft compared to fresh cartilage tissue, we performed FACS analysis of cluster of differentiation (CD) markers related to cell phenotype and immune response. The results from the FACS flow cytometric analysis (Fig. 6) reveal that the stained cells are positive for chondrogenic markers CD44+ (hyaluronic acid receptor) and CD49a (collagen receptor), and negative for hematopoietic stem cell marker CD45 (Leukocyte Common Antigen). The absence of immunogenic cell lines (CD45^−/−^) in the T-LE Allograft confirms an immune response will not be elicited after transplantation of the T-LE Allograft. The presence of both CD49a and CD44 indicates the chondrogenic capacity of the cells isolated from both the T-LE Allograft and fresh articular cartilage [21, 22].

## Discussion

In this study, we characterized a novel cartilage repair allograft (the T-LE Allograft) at the cell and tissue levels for viability, cellular outgrowth, metabolism, regenerative cell signaling, and immunogenicity. It is becoming increasingly clear that these characteristics are critical for successful graft survival and integration into a chondral defect site [23]. The data presented here supports our hypothesis that T-LE Allograft, after 2 years of cryopreserved storage at − 80 °C, maintains the necessary viable cells, proper protein signaling, and tissue scaffold in order to facilitate a positive clinical outcome.

Our initial studies focused on characterizing the T-LE Allograft’s cell viability and proliferative capacity at the time of transplantation; both characteristics have been shown to be primarily responsible for the maintenance of donor articular cartilage health in the long term [10, 24]. In a study published by Cook et al. in 2016, the importance of cell viability greater than 70% was established as necessary in order to achieve the best clinical outcomes, whereby grafts with low cell viability (< 70%) frequently result in failure [10]. This viability threshold has limited the availability of fresh OCAs and previous attempts of cryopreservation have resulted in detrimental reductions in chondrocyte viability [25]. Freshly recovered articular cartilage showed an average viability of 98.83 ± 0.43% with a range of 98.00 to 99.50%. In comparison, our cryopreserved T-LE Allograft post-thaw was found to have an average viability of 94.97 ± 3.38% at its maximum shelf life of 2 years with a range of tested values from 86.67 to 98.67%; we note that all grafts tested surpassed the 70% viability threshold throughout their shelf life. This is a significant improvement from currently available cryopreserved OCAs; for instance, one commercially available allograft product maintains an average viability near the 70% threshold but with a highly dispersed viability range (54.5–88.5%) [26].

To assess the performance of the T-LE Allograft under a typical fixation method, we developed an in vitro model to mimic the fixation environment often utilized during surgical repair. During the T-LE Allograft explantation studies, in vitro graft functionality via PrestoBlue assay demonstrated an increase in cellular outgrowth overtime, which was additionally supported with Safranin-O staining and IF imaging of the explanted grafts. This 12-week study revealed that post-cryopreservation, healthy chondrocytes retained within the T-LE Allograft were not directed toward apoptosis indicating they maintained their metabolic and proliferative capacities. Furthermore, the Safranin-O and IF imaging suggests a healing mechanism for chondral defects, as living cells proliferate into the fibrin glue and begin to construct new ECM scaffolding, confirmed by the presence of new aggrecan and collagen II deposits, both integral proteins in cartilaginous tissues, within the surrounding fibrin glue [5]. In addition to new scaffold production, the T-LE Allograft maintains the entirety of the native structure of articular cartilage throughout the 2-year shelf life. This includes the articular surface (superficial zone) which contains tightly packed collagen fibers and a relatively high number of chondrocytes. The integrity of the articular surface is critical for the protection and maintenance of the deeper zones, and therefore restoring the superficial layer alone may enhance healing of the deep zone cartilage compared to surgical procedures like microfracture [5].

Tissue and cell signaling events through proper ECM composition represent an important feature of successful regeneration and integration of allograft materials. For the success of the novel T-LE Allograft, it is important to maintain a growth factor and matrix protein profile similar to fresh articular cartilage throughout its 2-year shelf life in order to facilitate successful tissue integration. The following growth factors and matrix proteins have been identified to play an important role in maintaining the function of healthy cartilage: b-FGF, TGF-β1, BMP7, PRG4, and sGAG (Table 1). These growth factors and matrix proteins help facilitate intercellular communication, proper interaction and dynamics within the scaffold, and stem cell migration following a microfracture procedure. Thus, for the grafts to remain clinically impactful, it is important to compare T-LE Allograft’s growth factor and matrix protein profile to the composition of healthy, fresh adult cartilage. The biochemical assays revealed the growth factor and matrix protein profiles of the T-LE Allograft were similar to unprocessed fresh cartilage, suggesting that long-term cryopreserved storage had no significant negative impact on bioactivity.

**Table 1:** The growth factors and matrix proteins evaluated in this study. The essential role in cartilage function and possible dysfunction when improperly expressed are explored

Growth factor/matrix proteins	Effects in cartilage	Improper expression leads to
b-FGF (Basic Fibroblast Growth Factor)	Provides the response to tissue injury, by increasing chondrocyte proliferation, collagen II deposition, while preventing tissue hypertrophy [27, 28].	Lack of response to injury, hypertrophy of cartilage and non-proliferating cells [28, 29]
PRG4 (Superficial Zone Protein)	Is the source of joint lubrication, which protects against Osteoarthritis (OA) and inflammation. Also slows growth factor expression [30–33].	Joints lose lubrication leading to chondrocyte death and irreversible OA [34, 35]
TGF-β1 (Transforming Growth Factor Beta 1)	Maintains PRG4 levels allowing it to protect against OA and promotes cellular chondrogenesis [36, 37].	The onset of OA and eventual osteochondral degeneration [38, 39]
BMP-7 (Bone Morphogenic Protein 7)	Promotes stem cell chondrogenesis and their production of PRG4 and hyaline cartilage [40–42].	Cartilage tissue degradation [43]
sGAG (sulfated glycosaminoglycans)	Central role in maintaining cartilage homeostasis while providing structure and biomechanical properties [44].	The onset of OA and cartilage [45]

The functional influence of these growth factors and matrix proteins was confirmed in vitro in the BM-MSC co-culture experiments. Proper cell signaling is vital for successful defect repair, as shown by fibrocartilage formation in microfracture procedure [46, 47]. Our data supports the use of the T-LE Allograft, used in conjunction with microfracture, to aid in the proper migration and differentiation of the BM-MSCs. Signaling was assessed with the BM-MSCs differentiation and migration assays where it was evident that the T-LE Allograft was capable of influencing the microenvironment of the BM-MSCs to induce chondrogenesis similar to fresh cartilage. Other factors such as TGF-β1, b-FGF, and BMP-7 are also known to induce BM-MSC chondrogenesis, and, as shown from our ELISA assays, these signals are also excreted from the T-LE Allograft, permitting BM-MSCs in culture to differentiate and expand into chondrocytes [47–49]. These studies suggest that in vivo, when the T-LE Allograft is combined with microfracture, BM-MSCs will be recruited to the defect site and directed toward chondrogenesis to produce healthy articular cartilage as opposed to fibrocartilage. These characteristics improve articular cartilage repair over microfracture alone by producing collagen II and restoring the defect.

Another important characteristic of the T-LE Allograft, as it is made from articular cartilage, is its avascular nature which does not illicit an immune response after transplantation causing less pain and inflammation compared to traditional OCA grafts, which still contain blood and lipid in the boney portions of the graft [50]. The chondrocytes assessed within the T-LE Allograft and fresh articular cartilage were found to have negligible expression, less than 1%, of CD45+ an immunogenic marker. These cells also expressed both CD44 and CD49a confirming their proliferative capacity and chondrogenic potential in vivo.

While the results reported here for the T-LE Allograft have demonstrated promising results in vitro with similarities to native fresh articular cartilage, recent clinical studies have also showed the in vivo efficacy of these grafts. For example, the T-LE Allograft has recently been used to repair chondral lesions in various joints such as the knee, hip, talus, and first metatarsal with excellent outcomes [51–54]. Treatment of articular cartilage lesions with the T-LE Allograft has demonstrated sustained positive clinical results proving to be an effective technique in treating osteochondral lesions of various sizes and locations [51, 52]. The studies support the effectiveness of the T-LE Allograft clinical outcomes with important cell- and tissue-level analysis, highlighting the critical nature of viable cells, scaffolding, and matrix signaling toward successful tissue integration and regeneration.

We note several limitations to the data and results presented here. One major limitation of this study was the availability of research consented donor tissue that met the necessary requirements of the study due to age, recovery, and processing time restrictions. A limited sample size may not have been as statistically powerful due to donor variability. Future studies would benefit from a larger donor population combined with a more detailed biochemical and biomechanical analysis of pre- and post-cryopreservation of the T-LE Allograft.

## Conclusions

The Cryopreserved, Thin Laser-Etched Osteochondral Allograft (T-LE Allograft) is a surgical option for cartilage replacement and repair with similar properties to healthy, fresh articular cartilage. The data presented in this study reveals that the long-term cryopreservation (2-year shelf life) of the T-LE Allograft maintains viable and metabolically active cells, retains necessary growth factors, and preserves the ECM scaffolding. The average viability of the T-LE Allograft was 94.97% with all samples testing viability greater than the 70% threshold which is necessary for positive clinical outcomes. The T-LE Allograft also contains the necessary factors for recruitment and differentiation of BM-MSCs into chondrocytes to improve outcomes when used in conjunction with microfracture procedure.

Cryopreservation of the T-LE Allograft mitigates the limited shelf life characteristic of commonly utilized fresh osteochondral grafts, greatly expanding mainstream use to patients and clinicians. Furthermore, our findings reveal that the original composition and graft integrity have been maintained throughout this novel cryopreservation process and over the extent of its 2-year shelf life at − 80 °C storage. Due to its ability to retain similarities to fresh native articular cartilage, the T-LE Allograft provides a readily available allograft solution to aid in the difficult task of articular cartilage replacement and repair.

## Supplementary Information

**Additional file 1:** **Supplementary Table 1:** Study Design: an outline of the studies performed and the sample size of each test with the main characteristic explored in each study. Details of methods and statistics described in the Methods section.

**Additional file 2:** **Supplementary Figure 1:** Experimental design for In Vitro Fixation Model (A), Migration (B) and Differentiation (C) Assays.

## Data Availability

The datasets used and/or analyzed during the current study are available from the corresponding author on reasonable request.

## Abbreviations

b-FGF: Basic Fibroblast Growth Factor
BM-MSC: Bone marrow-derived mesenchymal stem cells
BMP-7: Bone Morphogenic Protein 7
CD: Cluster of differentiation
CGM: Chondrocyte growth media
DMEM: Dulbecco’s modified Eagle medium
ELISA: Enzyme-linked immunosorbent assay
FACS: Fluorescently activated cell sorting
FBS: Fetal bovine serum
FITC: Fluorescein isothiocyanate
H&E: Hematoxylin and eosin
IF: Immunofluorescent
OCA: Osteochondral allograft
PE: Phycoerythrin
SD: Standard deviation
SEM: Standard error of the mean
sGAG: Sulfated glycosaminoglycans
TGF-b: Transforming Growth Factor Beta
T-LE Allograft: Cryopreserved, Thin, Laser-Etched Osteochondral Allograft
PBS: Phosphate-buffered saline
PRG-4: Proteoglycan 4 (Superficial Zone Protein)

## References

[1] Hjelle K, Solheim E, Strand T, Muri R, Brittberg M (2002). Articular cartilage defects in 1,000 knee arthroscopies. Arthroscopy.

[2] Curl WW, Krome J, Gordon ES, Rushing J, Smith BP, Poehling GG (1997). Cartilage injuries: a review of 31,516 knee arthroscopies. Arthroscopy..

[3] Heir S, Nerhus TK, Røtterud JH (2010). Focal cartilage defects in the knee impair quality of life as much as severe osteoarthritis: a comparison of knee injury and osteoarthritis outcome score in 4 patient categories scheduled for knee surgery. Am J Sports Med.

[4] Strauss EJ, Fonseca LE, Shah MR, Yorum T (2011). Management of focal cartilage defects in the knee: is ACI the answer?. Bull NYU Hosp Jt Dis.

[5] Sophia Fox AJ, Bedi A, Rodeo SA (2009). The basic science of articular cartilage: structure, composition, and function. Sports Health..

[6] Steinert AF, Ghivizzani SC, Rethwilm A, Tuan RS, Evans CH, Nöth U (2007). Major biological obstacles for persistent cell-based regeneration of articular cartilage. Arthritis Res Ther..

[7] Sherman SL, Garrity J, Bauer K, Cook J, Stannard J, Bugbee W (2014). Fresh osteochondral allograft transplantation for the knee: current concepts. J Am Acad Orthop Surg..

[8] Gortz S, Bugbee W (2006). Allografts in articular cartilage repair. J Bone Joint Surg Am Vol.

[9] LaPrade RF, Botker J, Herzog M, Agel J (2009). Refrigerated osteoarticular allografts to treat articular cartilage defects of the femoral condyles. A prospective outcomes study. J Bone Joint Surg Am.

[10] Cook JL, Stannard JP, Stoker AM, Bozynski CC, Kuroki K, Cook CR, Pfeiffer FM (2016). Importance of donor chondrocyte viability for osteochondral allografts. Am J Sports Med..

[11] Capeci CM, Turchiano M, Strauss EJ, Youm T (2013). Osteochondral allografts: applications in treating articular cartilage defects in the knee. Bull Hosp Jt Dis.

[12] Gao L, Orth P, Cucchiarini M, Madry H (2019). Autologous matrix-induced chondrogenesis: a systematic review of the clinical evidence. Am J Sports Med.

[13] Makris EA, Gomoll AH, Malizos KN, Hu JC, Athanasiou KA (2015). Repair and tissue engineering techniques for articular cartilage. Nat Rev Rheumatol..

[14] Saris D, Price A, Widuchowski W (2014). Matrix-applied characterized autologous cultured chondrocytes versus microfracture: two-fear follow-up of a prospective randomized trial. Am J Sports Med.

[15] Xing L, Jiang Y, Gui J (2013). Microfracture combined with osteochondral paste implantation was more effective than microfracture alone for full-thickness cartilage repair. Knee Surg Sports Traumatol Arthrosc..

[16] Furukawa T, Eyre DR, Koide S, Glimcher MJ (1980). Biochemical studies on repair cartilage resurfacing experimental defects in the rabbit knee. J Bone Joint Surg Am Vol.

[17] Liu J, Liu X (2012). Conditioned medium from chondrocyte/scaffold constructs induced chondrogenic differentiation of bone marrow stromal cells. Anat Rec (Hoboken)..

[18] Mccormick F, Yanke A, Provencher MT, Cole BJ (2008). Minced articular cartilage—basic science, surgical technique, and clinical application. Sports Medicine and Arthroscopy Review..

[19] Beer AJ, Tauro TM, Redondo ML, Christian DR, Cole BJ, Frank RM. Use of allografts in orthopaedic surgery: safety, procurement, storage, and outcomes. Orthop J Sports Med. 2019;7(12). 10.1177/2325967119891435. 10.1177/2325967119891435 PMC6937533 31909057

[20] Lightfoot A, Martin J, Amendola A (2007). Fluorescent viability stains overestimate chondrocyte viability in osteoarticular allografts. Am J Sports Med.

[21] Grogan SP, Barbero A, Diaz-Romero J, Cleton-Jansen AM, Soeder S, Whiteside R (2007). Identification of markers to characterize and sort human articular chondrocytes with enhanced in vitro chondrogenic capacity. Arthritis & Rheumatism..

[22] Rousche KT, Knudson CB (2002). Temporal expression of CD44 during embryonic chick limb development and modulation of its expression with retinoic acid. Matrix Biology..

[23] Madeira C, Santhagunam A, Salgueiro JB, Cabral JM (2015). Advanced cell therapies for articular cartilage regeneration. Trends Biotechnol.

[24] Redondo ML, Naveen NB, Liu JN, Tauro TM, Southworth TM (2018). Cole BJ. Preservation of knee articular cartilage, sports medicine and arthroscopy review..

[25] Abazari A, Jomha NM, Elliott JA, McGann LE (2013). Cryopreservation of articular cartilage. Cryobiology.

[26] Geraghty S, Kuang J, Yoo D (2015). A novel, cryopreserved, viable osteochondral allograft designed to augment marrow stimulation for articular cartilage repair. J Orthop Surg Res.

[27] Chua KH, Aminuddin BS (2007). Basic fibroblast growth factor with human serum supplementation: enhancement of human chondrocyte proliferation and promotion of cartilage regeneration. Singapore Med J.

[28] Pangborn CA, Athanasiou KA (2005). Growth factors and fibrochondrocytes in scaffolds. J Orthop Res..

[29] Loeser RF, Chubinskaya S, Pacione C, Im HJ (2005). Basic fibroblast growth factor inhibits the anabolic activity of insulin-like growth factor 1 and osteogenic protein 1 in adult human articular chondrocytes. Arthritis Rheum..

[30] Muddasani P, Norman JC (2007). Basic fibroblast growth factor activates the MAPK and NFkappaB pathways that converge on Elk-1 to control production of matrix metalloproteinase-13 by human adult articular chondrocytes. J Biol Chem..

[31] Alquraini A, Garguilo S (2015). The interaction of lubricin/proteoglycan 4 (PRG4) with toll-like receptors 2 and 4: an anti-inflammatory role of PRG4 in synovial fluid. Arthritis Res Ther..

[32] Jay GD, Waller KA (2014). The biology of lubricin: near frictionless joint motion. Matrix Biol.

[33] Ruan MZ, Erez A, et al. Proteoglycan 4 expression protects against the development of osteoarthritis. Sci Transl Med. 5(176):176ra134. 10.1126/scitranslmed.3005409. 10.1126/scitranslmed.3005409 PMC3804124 23486780

[34] Chan SM, Neu CP (2010). Atomic force microscope investigation of the boundary-lubricant layer in articular cartilage. Osteoarthritis Cartilage.

[35] Waller KA, Zhang LX (2013). Role of lubricin and boundary lubrication in the prevention of chondrocyte apoptosis. Proc Natl Acad Sci U S A..

[36] Blaney Davidson EN, Scharstuhl A (2005). Reduced transforming growth factor-beta signaling in cartilage of old mice: role in impaired repair capacity. Arthritis Res Ther.

[37] Van der Kraan PM (2014). Age-related alterations in TGF beta signaling as a causal factor of cartilage degeneration in osteoarthritis. Biomed Mater Eng.

[38] Finnson KW, Chi Y, Bou-Gharios G, Leask A, Philip A (2012). TGF-b signaling in cartilage homeostasis and osteoarthritis. Front Biosci.

[39] Zhen G, Cao X (2014). Targeting TGFbeta signaling in subchondral bone and articular cartilage homeostasis. Trends Pharmacol Sci.

[40] Kuo AC, Rodrigo JJ (2006). Microfracture and bone morphogenetic protein 7 (BMP-7) synergistically stimulate articular cartilage repair. Osteoarthritis Cartilage..

[41] Sekiya I, Tang T (2009). Periodic knee injections of BMP-7 delay cartilage degeneration induced by excessive running in rats. J Orthop Res..

[42] Hayashi M, Muneta T (2010). Intra-articular injections of bone morphogenetic protein-7 retard progression of existing cartilage degeneration. J Orthop Res..

[43] Abula K, Muneta T (2015). Elimination of BMP7 from the developing limb mesenchyme leads to articular cartilage degeneration and synovial inflammation with increased age. FEBS Lett..

[44] Mertz EL, Facchini M, Pham AT, Gualeni B, De Leonardis F, Rossi A, Forlino A (2012). Matrix disruptions, growth, and degradation of cartilage with impaired sulfation. The Journal of biological chemistry..

[45] Stubendorff JJ, Lammentausta E, Struglics A, Lindberg D, Heinegard D, Dahlberg LE (2012). Is cartilage sGAG content related to early changes in cartilage disease? Implications for interpretation of dGEMRIC. Osteoarthritis and Cartilage.

[46] Liu X, Sun H (2012). In vivo ectopic chondrogenesis of BMSCs directed by mature chondrocytes. Biomaterials.

[47] Darling EM, Athanasiou KA (2005). Growth factor impact on articular cartilage subpopulations. Cell Tissue Res..

[48] Kulyk WM, Rodgers BJ, Greer K, Kosher RA (1989). Promotion of embryonic chick limb cartilage differentiation by transforming growth factor-beta. Dev Biol..

[49] Kwon H, Paschos NK, Hu JC, Athanasiou K (2016). Articular cartilage tissue engineering: the role of signaling molecules. Cell Mol Life Sci..

[50] Demange M, Gomoll AH (2012). The use of osteochondral allografts in the management of cartilage defects. Curr Rev Musculoskelet Med..

[51] Beth ZC, Sachs B, Kruse D, Stone PA (2019). Arthroscopic implantation of a cartilage matrix for an osteochondral defect of the talus: a case report. J Foot Ankle Surg..

[52] Mehta V, Mandala C, Shriver R. Clinical results of a novel 3D fresh cartilage matrix for focal articular cartilage defects. In: ICRS Summit 2019. San Diego: ICRS- International Cartilage Regeneration and on and Joint Preservation Society; 2019. https://cslide.ctimeetingtech.com/icrs2019/attendee/eposter/browse/gallery?q=mehta.

[53] Rao NM, Sachs BD, Kruse DL, Stone PA. The use of a cryopreserved fresh osteochondral allograft for repair of an osteochondral defect of the first metatarsal heal: a case presentation. In: American College of Foot and Ankle Surgeons (ACFAS) Annual Scientific Conference. New Orleans: American College of Foot and Ankle Surgeons; 2019. https://www.acfas.org/search.aspx#stq=Rao&stp=1.

[54] Faucett S, Scully R, Tomido S. Arthroscopic osteochondral allograft transplantation for focal grade 4 acetabular chondromalacia. In: ISHA Annual Scientific Meeting 2018. Melbourne: ISHA-The Hip Preservation Society; 2018. https://www.allosource.org/wp-content/uploads/2019/10/Faucett-Poster-ISHA.pdf.

