# Supplementary material for: Cryopreserved, Thin, Laser-Etched Osteochondral Allograft maintains the functional components of articular cartilage after 2 years of storage
Source: J Orthop Surg Res. 2020 Nov 11;15:521. doi: 10.1186/s13018-020-02049-y (PMC7659100; doi:10.1186/s13018-020-02049-y)
Supplement: Supplementary file 1 — Additional file 1: Supplementary Table 1: Study Design: an outline of the studies performed and the sample size of each test with the main characteristic explored in each study. Details of methods and statistics described in the Methods section. [file 13018_2020_2049_MOESM1_ESM.pdf]

**Study Title**	**Sample Size**	**Characterization**
Tissue Digest / Trypan Blue Exclusion Assay	Fresh Cartilage: 9 Donors [1 Sample per Donor]<br>Cryopreserved T-LE Allograft: 4 Donors [2-4 Samples per Donor]	Cellular Viability
Live/Dead Immunofluorescent Staining	Cryopreserved T-LE Allograft: 1 Donor [3 Samples per Donor]	Cellular Viability
Tissue Explant / Presto Blue Metabolic Assay	Fresh T-LE Allograft: 3 Donors [4-7 Samples per Donor]<br>Cryopreserved T-LE Allograft: 3 Donors [3 Samples per Donor]	Cellular Outgrowth and Metabolic Activity
ECM Immunofluorescent Staining	Fresh Cartilage: 1 Donor [2 Samples per Donor]<br>Cryopreserved T-LE Allograft: 1 Donor [2 Samples per Donor]	Presence of ECM Proteins
Histology: Structural Elements	Fresh Cartilage: 1 Donor [3 Samples per Donor]<br>Cryopreserved T-LE Allograft: 1 Donor [3 Samples per Donor]	ECM Structure
Growth Factor and Matrix Protein ELISAs	<u>TGF-β1</u>: Fresh Cartilage: 9 Donors<br>Cryopreserved T-LE Allograft: 19 Donors<br><u>PRG-4</u>: Fresh Cartilage: 9 Donors<br>Cryopreserved T-LE Allograft: 18 Donors<br><u>BMP-7</u>: Fresh Cartilage: 9 Donors<br>Cryopreserved T-LE Allograft: 18 Donors<br><u>bFGF</u>: Fresh Cartilage: 8 Donors<br>Cryopreserved T-LE Allograft: 9 Donors<br><u>sGAG</u>: Fresh Cartilage: 8 Donors<br>Cryopreserved T-LE Allograft: 9 Donors	Growth Factor and Glycosaminoglycan Profile
BM-MSC Migration and Differentiation Assay	Fresh Cartilage: 3 Donors [2 Samples per Donor]<br>Cryopreserved T-LE Allograft: 3 Donors [2 Samples per Donor]	Cellular Migration and Differentiation
Immunogenicity	Fresh Cartilage: 1 Donor<br>Cryopreserved T-LE Allograft: 1 Donor	Potential for Immunogenic Response

Supplementary Table 1: Study Design: an outline of the studies performed and the sample size of each test with the main characteristic explored in each study. Details of methods and statistics described in the Methods section.
