# Supplementary figures and images for: Cryopreserved, Thin, Laser-Etched Osteochondral Allograft maintains the functional components of articular cartilage after 2 years of storage
Source: J Orthop Surg Res. 2020 Nov 11;15:521. doi: 10.1186/s13018-020-02049-y (PMC7659100; doi:10.1186/s13018-020-02049-y)

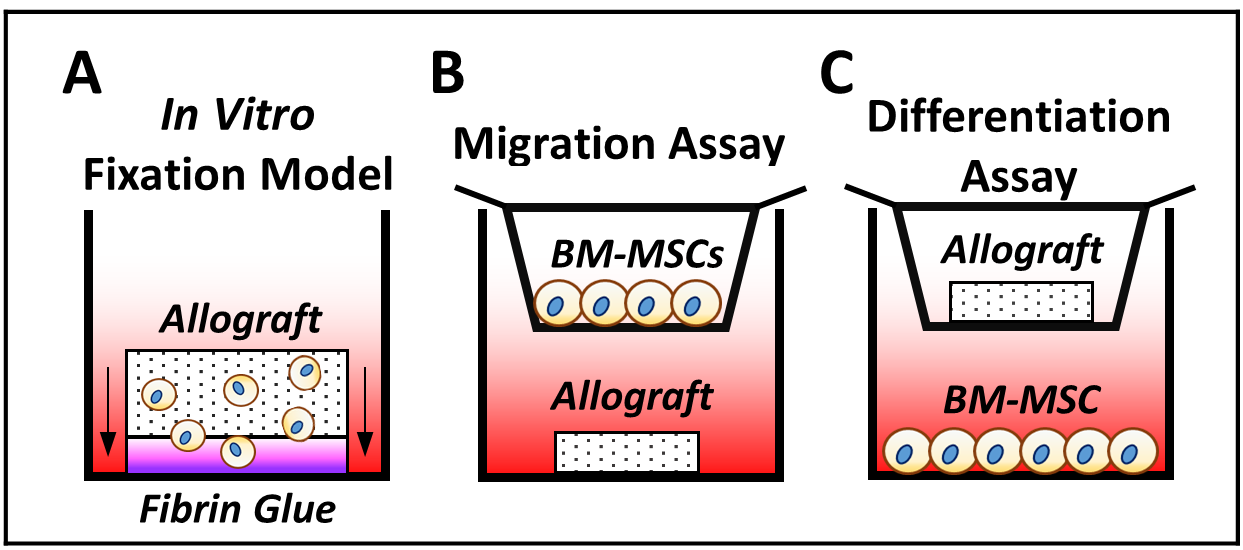

Supplement: Supplementary file 2 — Additional file 2: Supplementary Figure 1: Experimental design for In Vitro Fixation Model (A), Migration (B) and Differentiation (C) Assays. [file 13018_2020_2049_MOESM2_ESM.tif]
